# An 11p15 Imprinting Centre Region 2 Deletion in a Family with Beckwith Wiedemann Syndrome Provides Insights into Imprinting Control at CDKN1C

**DOI:** 10.1371/journal.pone.0029034

**Published:** 2011-12-19

**Authors:** Elizabeth Algar, Vinod Dagar, Menka Sebaj, Nicholas Pachter

**Affiliations:** 1 Molecular Oncology Laboratory, Murdoch Children's Research Institute, Melbourne, Australia; 2 Genetic Services of Western Australia, King Edward Memorial Hospital, Perth, Australia; 3 Department of Paediatrics, University of Melbourne, Melbourne, Australia; University of Bonn, Institut of Experimental Hematology and Transfusion Medicine, Germany

## Abstract

We report a three generation family with Beckwith Wiedemann syndrome (BWS) in whom we have identified a 330 kb deletion within the KCNQ1 locus, encompassing the 11p15.5 Imprinting Centre II (IC2). The deletion arose on the paternal chromosome in the first generation and was only associated with BWS when transmitted maternally to subsequent generations. The deletion on the maternal chromosome was associated with a lower median level of CDKN1C expression in the peripheral blood of affected individuals when compared to a cohort of unaffected controls (p<0.05), however was not significantly different to the expression levels in BWS cases with loss of methylation (LOM) within IC2 (p<0.78). Moreover the individual with a deletion on the paternal chromosome did not show evidence of elevated CDKN1C expression or features of Russell Silver syndrome. These observations support a model invoking the deletion of enhancer elements required for CDKN1C expression lying within or close to the imprinting centre and importantly extend and validate a single observation from an earlier study. Analysis of 94 cases with IC2 loss of methylation revealed that KCNQ1 deletion is a rare cause of loss of maternal methylation, occurring in only 3% of cases, or in 1.5% of BWS overall.

## Introduction

Beckwith Wiedemann syndrome (BWS) is an overgrowth disorder typically characterized by genomic imprinting defects on chromosome 11. Children affected by BWS have a broad spectrum of phenotypic features with varying degrees of severity including, pre and post-natal overgrowth, macrosomia, macroglossia, exomphalos, hemihypertrophy, cardiac defects, characteristic facial features and earlobe creases and pits. Children affected by BWS are at increased risk for cancer in the first seven years of life. Several distinct molecular sub-groups of BWS have been described including gain of methylation within the 11p15.5 Imprinting Centre 1 (IC1) leading to loss of imprinting of foetal IGF2 transcripts (5%), loss of methylation within the Imprinting Centre 2 (IC2) leading to CDKN1C silencing and biallelic expression of the antisense transcript, KCNQ1OT1 (50%), paternal uniparental disomy (15%), trisomy 11 p with paternal duplication and more rarely balanced translocations affecting maternal IC2, internal tandem duplications affecting paternal IC1 and inactivating mutations affecting maternal CDKN1C (5%) (reviewed in ([1])). Cancer risk is highest in patients with disruption to the imprinting or expression of foetal IGF2 caused by paternal uniparental disomy affecting both IC1 and IC2, structural abnormalities within IC1 including duplications and deletions, or gain of methylation on the maternal chromosome at IC1 [2], [3], [4], [5], [6], [7], [8], [9], [10].

Familial forms of BWS are rare and in all but one family described in the literature, inherited forms of BWS have involved the transmission of structural abnormalities affecting IC1 or CDKN1C mutation. There has only been one previous report of familial BWS with a deletion affecting IC2 [11]. In this family, maternal inheritance of the deletion was associated with BWS. The expression of CDKN1C examined in a single affected individual was at the lower end of the normal range consistent with a model of imprint regulation at IC2 involving cis-acting enhancer activation of maternal CDKN1C.

Imprint regulation within IC2 has not been as extensively studied as that within IC1. In mouse models, the paternal inheritance of targeted deletions within Kcnqt1, encompassing IC2, led to activation and biallelic expression of silenced alleles of genes including Cdkn1c, normally exclusively maternally expressed, consistent with a model involving obstruction of a silencing mechanism on the paternally inherited allele [12], [13], [14]. More recently it has been shown that the paternally expressed non coding RNA, kcnq1ot1, transcribed from a promoter 5′ with respect to kvdmr (the region of differential methylation within IC2), is directly involved in the bidirectional silencing in cis of paternal genes in the domain including Kcnq1, Cdkn1c, Ascl2, Cd81, and Osbp15. This is achieved through direct interaction between Kcnq1ot1 ncRNA, chromatin and the H3K9 and H3K27 histone methyl transferases G9a and the PRC2 complex [15], [16]. Additional complexity in imprint regulation within IC2 is suggested by observations of tissue specificity in imprint maintenance specifically affecting the Cdkn1c locus, and by the identification of insulator elements binding CTCF on the unmethylated paternal allele at a distance downstream from the Kcnq1ot1 promoter [17], [18]. This repressor activity facilitated by CTCF binding to the unmethylated paternal kvdmr is completely separable from the Kcnq1ot1 promoter, is independent of Kcnq1ot1 transcription, and suggests that the kvdmr may function as a chromatin insulator in a similar manner to the H19DMR within IC1. In the chromatin insulator model, differential methylation at the insulator maintains the imprinted expression of flanking genes by blocking the access of enhancer elements to gene promoters. However this model invokes the location of enhancer elements and gene promoters being on opposing sides of the DMR or insulator. Support for this model is suggested by the work of John et al [19] where it was shown that by inserting Cdkn1c transgenes with associated Kcnq1 flanking sequence in mice, the elements required for maternal specific Cdkn1c expression were located at a distance from the Cdkn1c promoter and indeed 3′ and beyond the kvdmr. Moreover, evidence was shown for separate enhancer elements regulating the expression of Cdkn1c in different tissues and in placenta. The distant enhancer model is consistent with the low level of CDKN1C expression demonstrated in a single BWS patient with deletion of the entire KCNQ1OT1 locus on the maternal chromosome, in which the putative enhancer is also presumably deleted [11]. However more data from human studies is required to validate this single observation and consolidate support for an enhancer model.

Families with BWS provide unique opportunities to examine the direct impact of imprinting centre deletions on phenotype and to validate studies in animals where imprinting control models have been tested [7], [9].We describe here a three-generation family with a deletion of the KCNQ1 and KCNQ1OT1/LIT1 locus (IC2) that arose de novo on a paternally inherited chromosome and was subsequently transmitted maternally through two generations. Two offspring in the second generation, had maternally inherited deletions, and were affected by BWS. One child in the third generation inherited the deletion from her affected mother and was more severely affected. CDKN1C expression studies on peripheral blood showed that the individuals with a maternally inherited deletion had, on average, lower levels of CDKN1C in their peripheral blood mononuclear cells when compared with family members without a deletion and normal controls. CDKN1C expression levels in deletion cases were comparable to levels in BWS cases with LOM affecting KvDMR within IC2. This study therefore provides important further validation of an imprinting control model for CDKN1C in humans involving distant enhancers. Importantly the maternal grandmother, carrying a de novo paternal IC2 deletion, did not have elevated levels of CDKN1C expression in her blood and nor did she have evidence of growth restriction consistent with Russell Silver syndrome suggesting that both CDKN1C silencer and enhancer elements were removed by the deletion. Her normal phenotype also supports findings in the previous family described in the literature in which it was proposed that the ncRNA KCNQ1OT1 may be redundant for normal development [11]. This is only the second report of familial BWS with disruption to IC2 described to date.

## Results

### Identification of an 11p15.5 IC2 deletion affecting three generations

**Figure 1:**
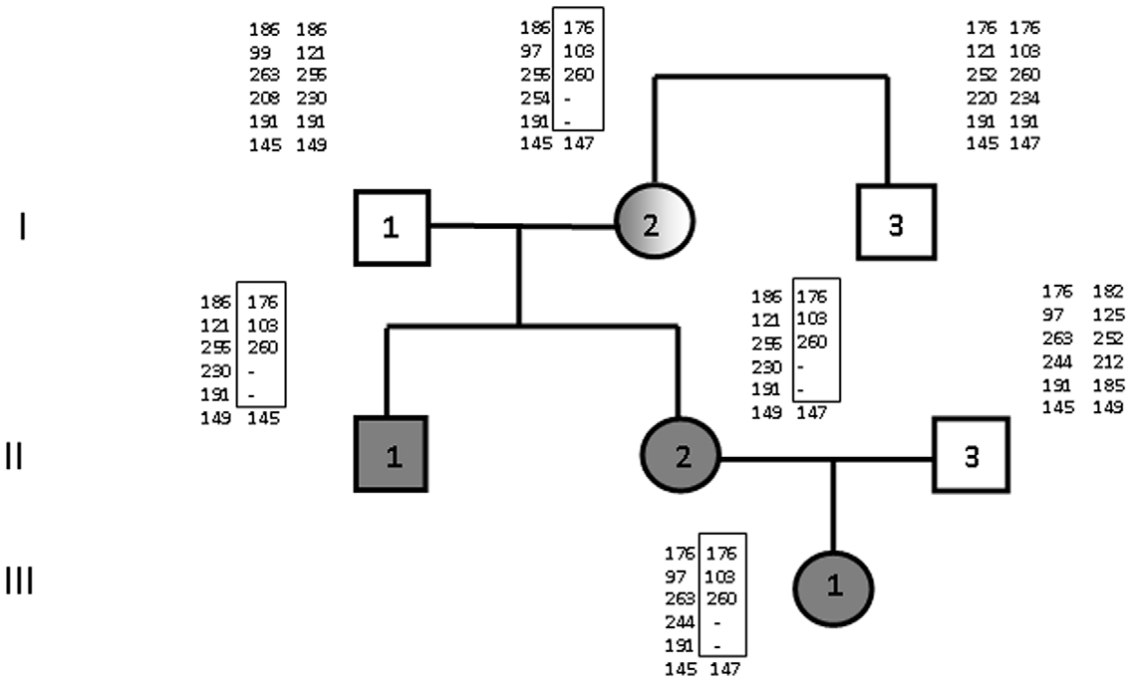
Pedigree of the three generation family showing individual 11p haplotypes. Affected individuals are indicated by full shading, carriers by partial shading. Haplotypes are represented by the allele sizes for each marker in descending order (telomere to centromere), *D11S576, D11S922, TH, D11S4088, 244.14* and *HBB*. Inferred marker deletion is indicated by a dash.

The family that is the subject of this article was identified following routine diagnostic testing for BWS in the female proband (III-1). The pedigree is shown in Figure 1. At birth she presented with exomphalos, macroglossia and transient hypogycaemia. Her birth weight was 3.51 Kg placing her on the 75^th^ percentile. She also had a naevus flammeus on her forehead, coarse facial appearance with an upturned nose and a right ear lobe crease. Subsequent growth and development were normal and she showed no evidence of hemihypertrophy. Further clinical investigation of the family revealed that her mother (II-2) and maternal uncle (II-1) both had features of BWS. The proband's mother had macroglossia at birth, a naevus flammeus over her forehead and an umbilical hernia. She did not develop hemihypertrophy and her birthweight was 3.03 kg (25^th^ percentile). Her development was normal. The maternal uncle was born prematurely and presented at birth with macroglossia, naevus flammeus over his forehead, and had hypoglycaemia as a neonate. His development was normal. The maternal grandmother, maternal grandfather and the maternal great uncle were all phenotypically normal, as was the proband's father. There was no history of malignancy in the individuals affected by BWS.

**Figure 2:**
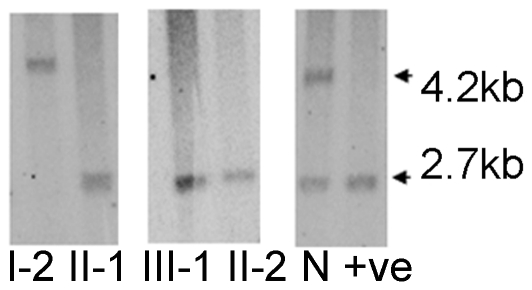
Southern blotting examining methylation at a Not1 site within KvDMR. The methylated maternal allele is at 4.2 kb and the unmethylated paternal allele is at 2.7 kb. Loss of the methylated maternal allele is shown for the affected individuals, II-1, II-2 and III-1. The methylated maternal allele is retained in I-2 and the unmethylated paternal allele is lost. Control DNA is represented by N and a positive control with loss of maternal allele methylation is in the adjacent lane.

11p15.5 methylation analysis was performed on DNA isolated from peripheral blood in the proband and then subsequently in all family members using standard methylation sensitive southern blotting at a Not1 site within the IC2 KvDMR [20], [21]. This analysis revealed a paternal only epigenotype in the proband and in her mother and maternal uncle, with a 2.7 kb band present on southern blotting (Figure 2). The maternal grandmother however displayed a maternal-only epigenotype and a 4.2 kb band. Real time karyotyping across the IGF2, KCNQ1 and CDKN1C loci was performed as described in [11]. All the affected family members and the maternal grandmother had reduced copy number for amplicons representing intron 1C, KCNQ1OT1 and exon 11 within the KCNQ1 locus, and maintained normal copy number at CDKN1C and IGF2 (Table 1). This analysis was subsequently followed by methylation sensitive multiplex ligation-dependant probe amplification (MS-MLPA) in the 11p15.5 region on both the affected and unaffected family members. MS-MLPA confirmed both the copy number change within KCNQ1 and the methylation change within KvDMR in the proband, her mother and uncle, and in the maternal grandmother (Table 2). IC1 methylation and copy number were normal in both affected and unaffected individuals. In all the individuals carrying the KCNQ1 deletion, exons 1 Alt and exon 16 were intact, suggesting it extends from KCNQ1 intron 1 to KCNQ1 intron 15, a distance of approximately 330 Kb. The methylation patterns observed on southern blotting and MS-MLPA are also consistent with a deletion within KCNQ1 on the paternal chromosome in the grandmother and subsequent transmission from the grandmother to her affected son and daughter and from her daughter to her grand-daughter.

**Table 1:** Q-PCR determination of copy number.

Case	CDKN1C	KCNQ1 intron 1C	KCNQ1OT1	KCNQ1 exon 11	IGF2
III-3	0.98	0.53	0.50	0.45	0.94
II-2	1.02	0.54	0.52	0.46	1.08
II-1	0.96	0.50	0.43	0.40	0.99
I-2	0.96	0.51	0.46	0.54	0.93
I-1	0.89	1.01	0.90	1.28	1.09

Relative gene copy number analysis for amplicons across the KCNQ1 and flanking loci determined by Q-PCR. The heading KCNQ1OT1 refers to DNA within the KCNQ1OT1 region. The values shown are the ratio of the means of triplicate copy number determinations relative to the mean of the CFTR reference gene copy number. Deletion within the KCNQ1 locus is suggested by reduced copy number in the DNA from I-2, II-1, II-2 and III-1. Normal copy number is maintained in I-1.

**Table 2:** Methylation sensitive MLPA.

Probe	Normal range	I-1	I-2	1-3	II-1	II-2	II-3	III-1
Copy No probe								
H19 2219	0.87–1.09	0.98	0.98	0.90	0.86	0.96	0.99	0.91
H19 10585	0.79–1.19	0.92	1.05	0.97	0.99	1.03	0.98	0.91
H19 10586	0.87–1.09	0.92	0.86	1.01	0.83	0.87	0.96	0.98
H19 6268 (exon 3)	0.91–1.03	1.04	1.06	1.03	1.02	0.98	1.00	0.97
H19 10588	0.77–1.23	0.86	0.94	0.91	0.90	1.01	0.86	0.842
IGF2 6272 (exon 3)	0.88–1.08	0.99	1.00	0.95	0.98	0.92	1.00	0.95
KCNQ1 3537 (Alt exon 1)	0.97–1.07	0.94	0.96	0.92	1.09	1.03	0.93	0.92
KCNQ1 3539 (exon 3)	0.85–1.10	0.99	**0.51**	1.01	**0.54**	**0.56**	0.99	**0.53**
KCNQ1 3542 (exon 6)	0.87–1.16	0.96	**0.55**	0.95	**0.54**	**0.55**	0.95	**0.54**
KCNQ1 3543 (exon 7)	0.83–1.25	1.05	**0.50**	1.00	**0.54**	**0.54**	1.00	**0.54**
KCNQ1 3544 (exon 8)	0.93–1.13	1.02	**0.51**	0.98	**0.56**	**0.52**	0.99	**0.55**
KCNQ1 3550 (exon 12)	0.90–1.04	0.99	**0.49**	1.00	**0.53**	**0.52**	1.01	**0.53**
KCNQ1 3553 (exon 15)	0.88–1.14	1.01	**0.53**	0.99	**0.52**	**0.55**	0.98	**0.58**
KCNQ1 3555 (exon 16)	0.90–1.04	0.99	0.90	0.97	0.97	0.98	0.94	0.98
CDKN1C 6262 (exon 1)	0.93–1.05	1.02	0.92	0.97	0.97	0.97	0.99	0.99
CDKN1C 6263 (exon 1)	0.96–1.18	0.98	0.94	1.03	0.91	0.92	0.98	0.96
Methylation probe								
H19 DMR 8743	0.52–0.62	0.55	0.50	0.53	0.57	0.57	0.51	0.62
H19 DMR 8744	0.53–0.63	0.61	0.56	0.60	0.57	0.58	0.61	0.52
H19 DMR 11080	0.41–0.63	0.53	0.56	0.54	0.49	0.55	0.54	0.48
H19 DMR 6266	0.40–0.54	0.49	0.51	0.50	0.52	0.50	0.50	0.51
KvDMR 7173	0.53–0.63	0.57	**1.01**	0.60	**0.00**	**0.00**	0.62	**0.00**
KvDMR 6267	0.48–0.57	0.52	**0.88**	0.60	**0.00**	**0.00**	0.55	**0.00**
KvDMR 7171	0.51–0.67	0.61	**0.94**	0.57	**0.00**	**0.00**	0.54	**0.00**
KvDMR 7172	0.48–0.61	0.53	**0.96**	0.54	**0.00**	**0.00**	0.53	**0.00**

MS-MLPA analysis of the pedigree showing deletion from exon 2 to exon 15 within KCNQ1 in I-2, II-1, II-2 and III-1 (bolded), and maintenance of normal copy number in the remaining unaffected individuals. Normal methylation was maintained at the H19DMR (IC1) in all individuals and KvDMR methylation is abnormal in I-2, II-1, II-2 and III-1 (bolded). In I-2 the KvDMR methylation values are indicative of retention of the methylated IC2 on the maternal KCNQ1 allele and loss of the paternal unmethylated IC2, and in II-1, II-2 and III-3 these values are consistent with loss of the methylated IC2 on maternal KCNQ1 and retention of the paternal unmethylated IC2.

### Haplotype analysis suggests the IC2 deletion arose de novo

Haplotype analysis at markers mapping to 11p15 was performed on all family members using methods previously described in Algar et al [2]. This revealed evidence of the inheritance of a stable haplotype block within the family extending from D11S576 located 0.245 Mb from the telomere (Chromosome 11 Build 37.1) to a region located distally with respect to the HBB locus. The haplotype carrying the KCNQ1 deletion identified in the grandmother is presumed on the basis of methylation analysis to be derived from her father. However her brother shares the same haplotype for the markers TH, D11S922 and D11S576, located telomeric with respect to KCNQ1, and he does not carry a KCNQ1 deletion. This strongly suggests that the KCNQ1 deletion has arisen de novo in the grandmother on her paternal chromosome. The grandmother was phenotypically normal, with no remarkable features, suggesting that the removal of functional silencing elements within IC2, shown to be active on the paternal chromosome in mouse models, and KCNQ1OT1 transcription on the paternal chromosome, may not be essential for normal human development within the context of a deletion of this size. Only individuals inheriting the deletion maternally are affected by BWS.

### CDKN1C expression is reduced in IC2 deletion and KvDMR loss of methylation BWS cases

**Figure 3:**
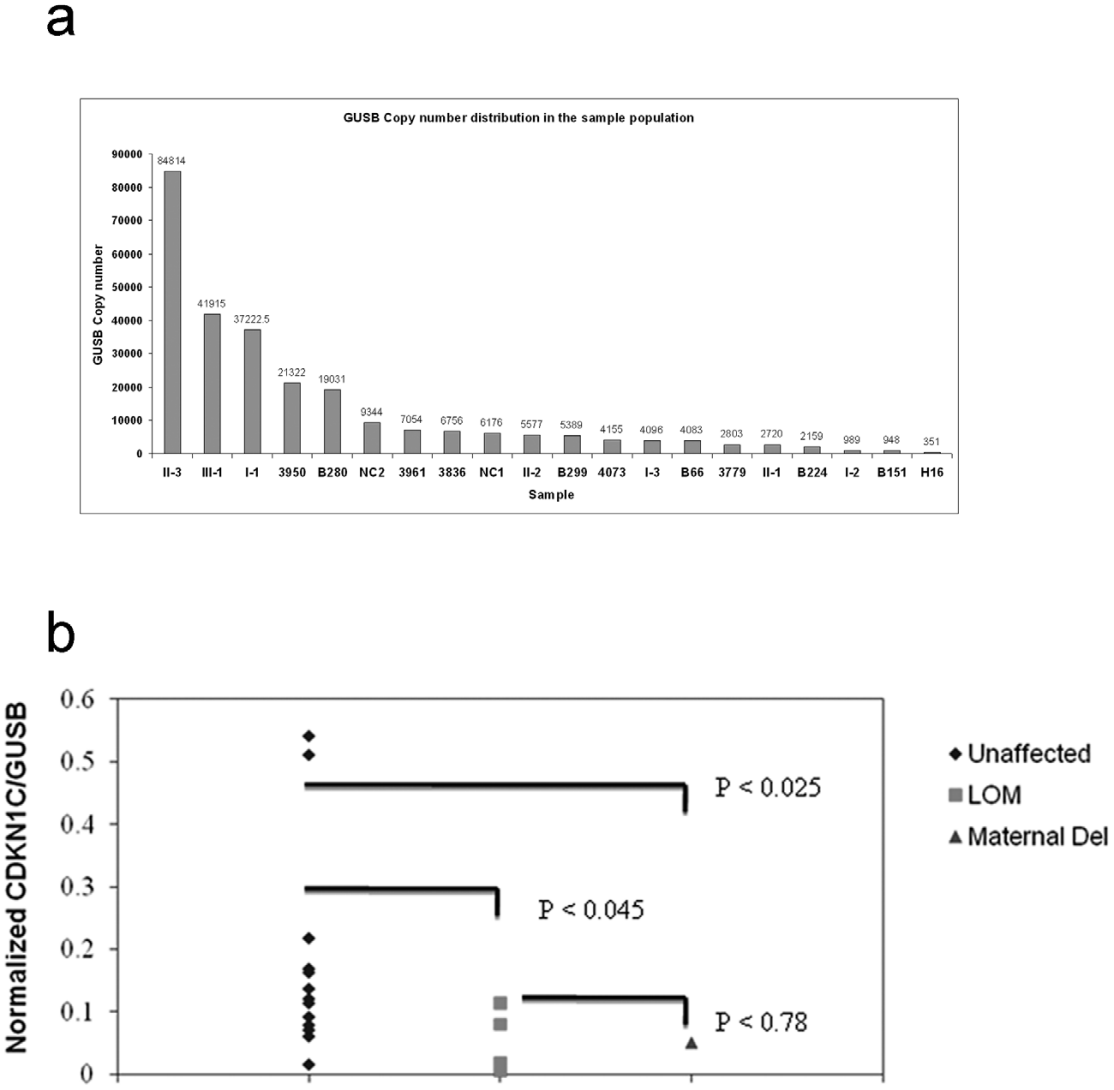
GUSB copy number distribution in the BWS and control population. (a) GUSB expression values were obtained from a 50 ng equivalent of total RNA. Quantitative analysis of CDKN1C expression. (b) CDKN1C expression in the blood from 12 unaffected individuals (diamonds), five BWS cases with loss of methylation at IC2 and no deletion (squares), and individuals with deletion of the maternal IC2 (triangles). The Mann Whitney non-parametric U test was used to derive the P values shown. Statistically significant P values were obtained for the population differences in CDKN1C expression between unaffected controls and cases with loss of methylation and KCNQ1/IC2 deletion.

CDKN1C expression was measured in the peripheral blood mononuclear cells of all family members depicted in Figure 1. CDKN1C copy number was determined and expressed relative to the GUSB copy number derived in cDNA samples from each individual, from normal controls and for comparison, from BWS cases with IC2 loss of methylation (LOM). Although the distribution of GUSB copy number in the sample population was broad, GUSB expression has been shown in previous validation studies to reliably reflect cDNA quantity for normalized gene copy number determinations for monitoring minimal residual disease in leukaemia, and was selected on this basis [22]. Furthermore in this previous study a similar population distribution in GUSB copy number values was obtained (10^3^–10^5^ copies per 100 ng equivalent of RNA) similar to what is reported here. Mean GUSB copy number values measured in samples tested in this study are shown in Figure 3a. All three individuals in the family affected by BWS, III-1, II-1 and II-2 individually had mean CDKN1C/GUSB ratios of 0.05. This was calculated as the mean of RQ-PCR duplicates, from two cDNA preparations from each individual. The maternal grandmother (I-1), with a paternal deletion, had a CDKN1C level of 0.07. The remaining unaffected members of the family had relative CDKN1C levels ranging from 0.06 to 0.14. The range established in the normal population, through an analysis of 11 unaffected individuals, extended from 0.015 to 0.54, and in five BWS patients examined with KvDMR LOM, extended from 0.006 to 0.114. The Mann Whitney non-parametric U test was used to examine the statistical significance of the differences in CDKN1C expression between individuals with a deletion and those in the unaffected population, including the unaffected family members, and between individuals with KvDMR LOM and the unaffected population. This analysis measures the significance of the distribution of values in each population and compares median values. Statistically significant p values of <0.05 were obtained for the deletion and loss of methylation groups who had lower CDKN1C expression levels when compared as a group to the unaffected controls (Figure 3b). The difference in CDKN1C expression between KCNQ1 deletion and KvDMR LOM cases was however not statistically significant (p<0.78) suggesting that the distribution of CDKN1C expression in these individuals is highly similar with both groups having lower levels of CDKN1C expression in peripheral blood.

The proband in the family was heterozygous for a deletion polymorphism within the CDKN1C exon 1 coding region however all other individuals in the family, with a deletion, were homozygous for the CDKN1C wild-type (non-polymorphic) “a” allele. Numerous attempts to examine the pattern of allelic expression at CDKN1C in the proband, using gel-based product identification, failed following RT-PCR due to the very low level of CDKN1C expression and the low efficiency of the PCR across the GC-rich repeat polymorphic region within CDKN1C exon 1 [23]. It was also not possible to redesign primers to generate a shorter amplicon to examine allelic expression because of the highly repetitive sequence within this part of exon 1.

### KCNQ1 deletion is rare in BWS

All cases with BWS referred to the laboratory in whom isolated LOM at KvDMR was originally ascertained by methylation sensitive southern blotting (excluding cases with UPD and trisomy 11p15) were reexamined by either Q-PCR and MS-MLPA for evidence of copy number change within IC2. Of a total of 94 cases evaluated as having LOM within IC2, a deletion was not identified in any. Thus KCNQ1 deletion is a rare cause of an apparent IC2 methylation defect in BWS, occurring at a frequency of 3% (3/97) in IC2 LOM cases.

## Discussion

In studying this BWS family we have made several important observations that support and also extend the observations previously reported in a BWS family with an 11p15.5 IC2 deletion [11]. Firstly, although IC2 deletion is rare, this case highlights the importance of thoroughly evaluating family history in BWS and testing relatives in cases where a deletion is identified. The technique of MS-MLPA has made the identification of these cases a relatively straightforward exercise, since the copy number at eight probes within the KCNQ1 locus as well as the methylation status of four probes within the KvDMR is evaluated simultaneously. Secondly, this family demonstrates that large paternal KCNQ1 deletions may have no identifiable phenotype if they delete both putative silencing and enhancer mechanisms involved in the regulation of CDKN1C expression on the paternal chromosome. KCNQ1 deletion causes BWS only when transmitted maternally and causes the syndrome through its effect on maternal CDKN1C expression. Thirdly the identification of this family within the context of routine molecular testing for BWS has enabled us to predict with accuracy the frequency with which IC2 deletions occur within the population of BWS patients affected by loss of methylation.

**Figure 4:**
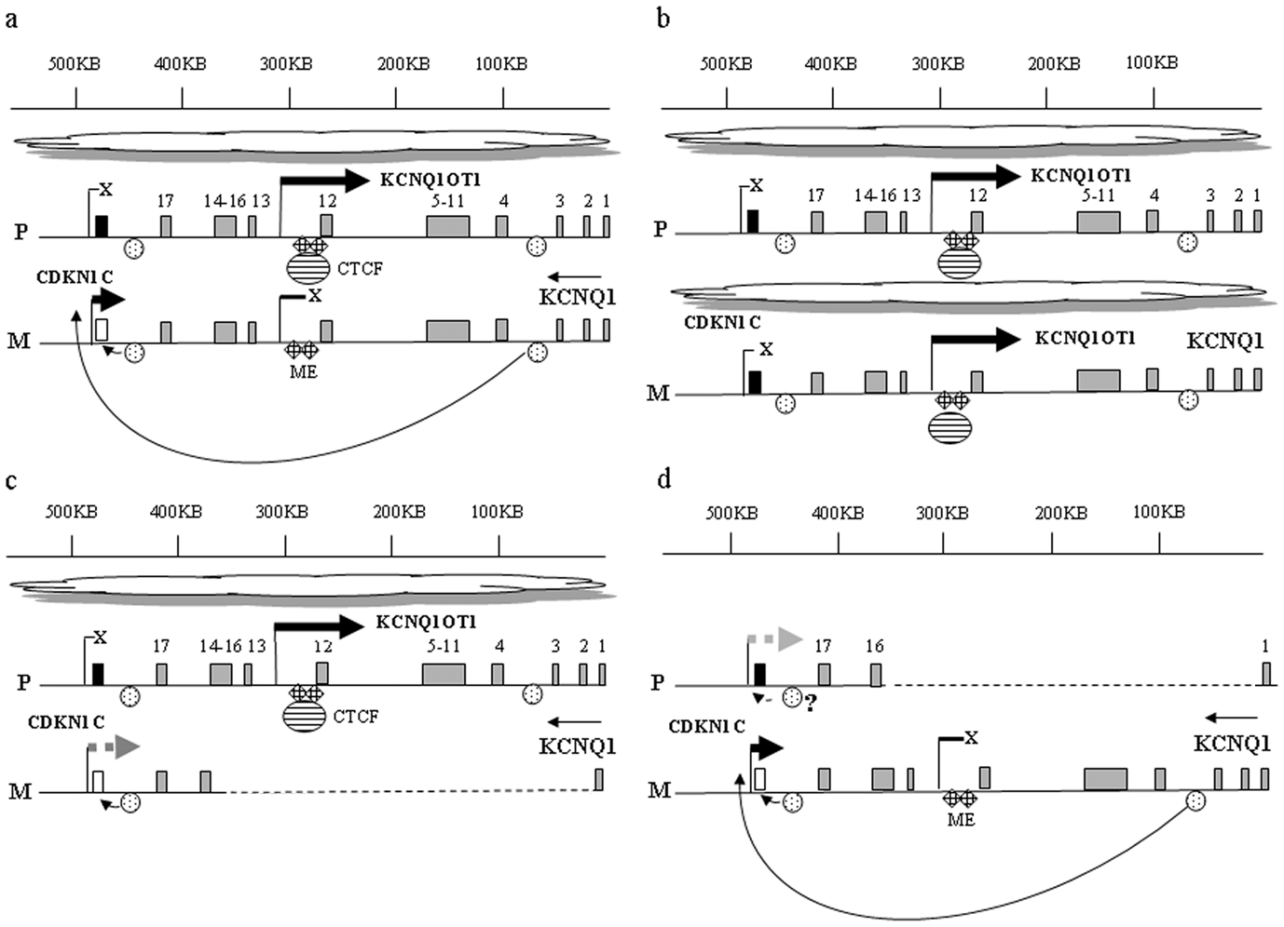
Model of normal and disrupted imprinting regulation at the CDKN1C locus. Genomic distances were calculated from NCBI reference sequence NG_008935.1. (a) CDKN1C imprinting is maintained on the paternal chromosome by KCNQ1OT1 transcription leading to bidirectional gene silencing (indicated by spreading cloud) and formation of a chromatin insulator within the KVDMR (CTCF binding) that prevents a dominant distant CDKN1C enhancer from activating paternal CDKN1C expression. On the maternal chromosome, methylation within the KVDMR prevents chromatin insulator formation, KCNQ1OT1 is silent, and the distance enhancer can activate maternal CDKN1C expression. (b). In BWS cases with LOM within KvDMR, bidirectional gene silencing on both paternal and maternal chromosomes occurs, chromatin insulators exist on the maternal and paternal chromosomes and distant CDKN1C enhancers can no longer activate maternal CDKN1C expression. Mosaicism for the LOM epimutation allows some CDKN1C expression to occur. (c). Maternal deletion of 330 kb within the KCNQ1 locus removes the KvDMR, and the distant enhancer element, thereby reducing the maternal CDKN1C expression that is normally activated by distant enhancers. Enhancers located close to CDKN1C, not affected by deletion, may still be active and allow CDKN1C expression in some tissues. (d). Paternal deletion of 330 kb has a minimal effect on CDKN1C expression because even though the KvDMR is deleted on the paternal chromosome, the distant enhancer for CDKN1C is also deleted, neutralizing any potential for activation of paternal CDKN1C. Proximal CDKN1C enhancers unaffected by deletion may be active in some tissues. Maternal CDKN1C expression is unaffected.

The evidence we show of significantly reduced CDKN1C expression in the peripheral blood of individuals with a maternal deletion, is compatible with a model invoking the deletion of enhancer elements within the KCNQ1 locus that are required for maternal CDKN1C expression. The enhancer model must however also be compatible with CDKN1C silencing observed in BWS patients with epigenetic defects within the KvDMR including loss of maternal methylation (LOM) described here and in Diaz-Meyer et al [24]. One possible explanation is that the induction of the expression of the ncRNA, KCNQ1OT1, on the maternal chromosome and its interactions with chromatin lead to bidirectional silencing in cis throughout the domain and that these changes also prevent maternal CDKN1C enhancer elements from functioning as a result of the altered chromatin structure. On the basis of our findings, putative enhancer elements required for CDKN1C expression must be located between KCNQ1 exons 3 and 15. This location could place them downstream (3′) of the insulator at KvDMR, thereby also satisfying a model based on an insulating DMR. Figure 4 presents a hypothetical model of how the KCNQ1OT1 antisense transcript, chromatin insulators and CDKN1C enhancers interact to maintain imprinted CDKN1C expression.

Although a CDKN1C promoter mutation might explain reduced CDKN1C expression in BWS cases with either loss of methylation within KvDMR or a KCNQ1 deletion, this was not considered to be a likely possibility given the rarity of CDKN1C mutation and the extremely unlikely scenario that two distinct constitutional mutations may have caused the syndrome in this instance. Furthermore previous studies have not demonstrated any evidence for co-occurrence of CDKN1C mutation with KvDMR loss of methylation.

Mice with targeted deletion within paternal KvDMR are 20–25% smaller than their wild-type littermates [12]. However the maternal grandmother in this family was not excessively short and did not show CDKN1C expression above the normal range in her blood. In fact her CDKN1C level was at the lower end of the normal range. The location of the large 330 kb deletion on her paternal chromosome may not lead to elevated CDKN1C expression if it deletes both paternal silencing as well as more distant enhancer elements that are required for CDKN1C expression (Figure 4). CDKN1C is not normally expressed from the paternal chromosome and silencing is thought to be maintained in part by antisense transcription of KCNQ1OT1 and by insulating elements. The deletions engineered in mice and in other cell models have generally been small and localized to the KvDMR region rather than encompassing flanking regions within the KCNQ1 locus, and therefore may not accurately predict the impact of larger KCNQ1 deletions on CDKN1C expression or phenotype. This is suggested by the observation that mice with targeted deletion of maternal KvDMR have no phenotype when the deletion is maternally transmitted [12]. Only paternal deletions that expose CDKN1C enhancers by opening up chromatin and that prevent KCNQ1OT1 transcription and disrupt formation of chromatin insulators, may lead to paternal CDKN1C expression and phenotypes linked to elevated CDKN1C.

This study is important for both clinicians involved in counseling for BWS and for researchers interested in imprinting control. Methylation within IC1 was unaltered by the presence of the KCNQ1 deletion, consistent with previous reports [11], [12], [25], however we were not able to examine allelic IGF2 expression directly due to the lack of IGF2 transcripts in peripheral blood. The cancer risk in KCNQ1 deletion cases of BWS is presently unknown due to the low number of affected individuals however BWS patients with isolated loss of methylation within IC2 are at risk for hepatoblastoma and other tumours including rhabdomyosarcoma, gonadoblastoma and thyroid carcinoma [6]. Whether the aetiology of these cancers is primarily attributable to the loss of CDKN1C expression in target tissues is currently uncertain, and it would be wise to maintain the tumour screening protocols in IC2 deletion cases, currently performed for loss of methylation cases.

## Materials and Methods

### Subjects

The family was recruited into the study following the identification of an IC2 deletion in the proband. Individuals gave written informed consent for participation and the study was performed with the approval of the Human Ethics Research Committee of the Royal Children's Hospital, approval EHRC 21121A. In the case of the infant proband, written informed consent was granted by the parents. DNA was isolated from peripheral blood using the Puregene Blood Core Kit B (Qiagen). RNA was isolated from peripheral blood using the RNeasy Kit (Qiagen) and 1.0 ug reverse transcribed to cDNA with random primers (pd(N)6) (Amersham Pharmacia Biotech) and M-MLV Reverse Transcriptase RNaseH minus (Promega Corporation).

### Genotyping

DNA was genotyped using microsatellite markers mapping to 11p. These were the dinucleotide repeats at D11S576 (0.245 M) and D11S922 (1 M) within 11p15.5, the tetranucleotide repeat region of Tyrosine Hydroxylase (TH) (2.19 M) at 11p15.5, the dinucleotide repeats D11S4088 (2.755 M) and 244.14 (2.68 M) within the KCNQ1 locus at 11p15.5, and the dinucleotide repeat of the HBB locus at 11p15.4 (5.22 M). Bracketed numbers represent the approximate location of markers on Chromosome Build 37.1. Primers were labelled with Fam, Hex or Tet and 15 ng of DNA was subjected to 35 cycles of PCR using a PTC-225 DNA Engine Tetrad (MJ Research). 1 uL PCR products from different reactions were pooled and electrophoresed on 4.5% 0.2 mm denaturing polyacrylamide gels on an ABI 377 DNA sequencer employing TAMRA 500 (Red) size standard. Sample lanes were tracked with Genescan software Version 3.1.2 and analysed using Genotyper Version 2.1.

### Southern blotting

Methylation at KvDMR (OMIM 604115) was examined by southern blotting following digestion of 5 ug DNA overnight at 37°C with 40 units each of EcoRI and NotI. Digested DNA was then electrophoresed on 1% gels overnight in 1XTAE buffer, and transferred in 20× SSC buffer onto Zeta Probe ^R^ GT Genomic tested blotting membrane (Biorad, Hercules CA 94547). Membranes were fixed under UV light, prehybridized at 60°C in ExpressHyb buffer (Clontech, Mountain View CA 94043) and hybridized with a ^32^P-labelled DMRP probe, recognizing a 4.2 kb methylated and 2.7 kb unmethylated band [21]Membranes were washed at room temperature with 2× SSC/0.05% SDS for 40 mins and then with 0.1× SSC/0.1% SDS for a further 40 mins at 55°C and exposed to phosphoimager screens for 24 to 48 hours. Bands were visualized with ImageQuant TL v2003.02 software following digital capture on a scanning phosphoimager (Molecular Dynamics). The methylation index was calculated as the peak volume of the methylated band divided by the sum of the volumes of the methylated and unmethylated bands after background subtraction. Reference ranges for normal methylation in the unaffected population were established from an analysis of 20 controls. These were determined as 0.52+/−0.17 (mean+/−2 SD). Pathological methylation was designated as methylation outside two standard deviations from the mean.

### Methylation sensitive multiplex ligation dependant probe amplification

200 ng DNA was subjected to methylation sensitive multiplex ligation dependant probe amplification (MS-MLPA) using the BWS/RSS ME030 kit (MRC Holland). Copy number data was analysed relative to a control DNA sample, comprising a mix of DNA from four unaffected individuals, after internal control normalization, and copy number was determined using GeneMarker software v 1.91 (Softgenetics). Methylation data was analysed relative to paired copy number samples run in parallel after internal control normalization, and methylation ratios calculated using the genomic imprinting analysis option in GeneMarker. Methylation ranges in the unaffected population, for each probe targeting methylation sensitive Hha1 sites within the 11p15.5 imprinting centres 1 and 2, were established by analyzing the DNA from 20 unaffected individuals. Pathological methylation was defined as methylation that was two standard deviations (SD) outside the normal population range at each probe, where the normal range was defined as the mean methylation value +/− (CVA+CVI+SD). For copy number, normal ranges were similarly determined. Any sample with an abnormal normalized copy number or methylation probe ratio was repeated. Methylation within an imprinting centre was not considered to be abnormal unless all methylation probes within the imprinting centre were ≥2 SD outside the normal range.

### Q-PCR karyotyping

Quantitative PCR across the IC2 was performed on DNA using similar methods to those described in Niemitz et al [11].Copy number within three regions of the KCNQ1 locus and at single regions with the CDKN1C and IGF2 loci were examined and normalized to the reference gene CFTR. Reaction mixes were comprised of a total volume of 25 ul and contained 12.5 ul of Fast Start Mastermix (Roche), 22.5 picomole of each primer, 3.1 picomoles of each hydrolysis probe and 5 ul of DNA. Standard curves were prepared for each gene analysed using DNA standards of 200 ng, 100 ng, 50 ng, 25 ng, 12.5 ng, 6.25 ng and 3.125 ng. Individual patient DNA samples were analysed at 50 ng. Reactions were run on a Rotorgene 3000 (Qiagen) for 50 cycles (50°C 2 mins, 95°C 10 mins and 50 cycles of 95°C for 15 secs, 60°C for 60 secs). Reaction runs included DNA standards, water non-template controls and individual DNA samples. Samples and standards were run in triplicate and Ct values were within 0.3 Ct units of each other. Standard curve gradients were within 3.32+/−0.30 when plotted against DNA concentration, and reaction efficiencies were in the range from 90 to100%.

Primer and hydrolysis probe sequences and fluorophores, for amplicons for copy number determination within the KCNQ1 locus (KCNQ1 intron 1C, KCNQ1OT1/KvDMR/LIT1, KCNQ1 exon 11), IGF2 and CDKN1C were as described in Niemitz et al [11]. Primers and probe to the reference sequence CFTR were: CFTR24F 5′ GAAGAGAACAAAGTGCGGCAG 3′, CFTR24R 5′ TTGCCGGAAGAGGCTCCT 3′and CFTRexon24 5′(HEX) ACGATTCCATCCAGAAACTGCTGAACGA (BHQ) 3′.

Relative copy number calculations were performed using the relative quantitation analysis (two standard curves) option in the Rotorgene software. The normal ranges (mean +/− SD) established in control samples for relative copy number for each amplicon were 0.99+/−0.03 (CDKN1C), 1.07+/−0.08 (KCNQ1 exon 11), 1.00+/−0.06 (KCNQ1 intron1C), 1.05+/−0.15 (KCNQ1OT1) and 1.01+/−0.022 (IGF2).

### CDKN1C expression

CDKN1C expression was examined by RQ-PCR on a Rotorgene 3000 (Qiagen). Primers and probes were as described in Niemitz et al [11]. Reaction mixes of 25 ul volume contained 12.5 ul of 2× Quantitect Probe master mix, 10picomole of each primer and 2.5 picomole of hydrolysis probe. Linearized CDKN1C plasmid standards were prepared by digestion of plasmid CDKN1C pcMV6-XL4 (Origene Technologies, Rockville, MD) with Kpn1. Linearized plasmid was diluted to generate six standards containing from 10^5^ to 1 plasmid copy number.

CDKN1C expression was normalized to the expression of the GUSB reference gene. Cycling conditions for GUSB expression were as described in Gabert et al [22]. GUSB standards for quantitation were prepared by serial dilution of GUSB plasmid linearized with Kpn1. Six standards in the range from 10^5^ to 1 plasmid copy were used in RQ-PCR. GUSB plasmid was prepared by cloning a 268 fragment encompassing exons 11 and 12 into PCR-Script Amp SK (+) cloning vector (Stratagene). Primer sequences used to generate a GUSB product from K562 leukaemia cell line cDNA for cloning were ex11F_1739 5′ctgatgttcactgaagagtacc 3′and ex12R_2007 5′ cattgtgacttggctactgagtg 3′. Standard curve gradients were 3.30+/0.10 (CDKN1C) and 3.34+/−0.04 (GUSB). Reaction efficiencies were close to100% for each reaction.

## References

[1] Enklaar T, Zabel BU, Prawitt D (2006). Beckwith-Wiedemann syndrome: multiple molecular mechanisms.. Expert Rev Mol Med.

[2] Algar EM, St Heaps L, Darmanian A, Dagar V, Prawitt D (2007). Paternally inherited submicroscopic duplication at 11p15.5 implicates insulin-like growth factor II in overgrowth and Wilms' tumorigenesis.. Cancer Res.

[3] Bliek J, Gicquel C, Maas S, Gaston V, Le Bouc Y (2004). Epigenotyping as a tool for the prediction of tumor risk and tumor type in patients with Beckwith-Wiedemann syndrome (BWS).. J Pediatr.

[4] DeBaun MR, Niemitz EL, McNeil DE, Brandenburg SA, Lee MP (2002). Epigenetic alterations of H19 and LIT1 distinguish patients with Beckwith-Wiedemann syndrome with cancer and birth defects.. Am J Hum Genet.

[5] Demars J, Shmela ME, Rossignol S, Okabe J, Netchine I Analysis of the IGF2/H19 imprinting control region uncovers new genetic defects, including mutations of OCT-binding sequences, in patients with 11p15 fetal growth disorders.. Hum Mol Genet.

[6] Weksberg R, Nishikawa J, Caluseriu O, Fei YL, Shuman C (2001). Tumor development in the Beckwith-Wiedemann syndrome is associated with a variety of constitutional molecular 11p15 alterations including imprinting defects of KCNQ1OT1.. Hum Mol Genet.

[7] Prawitt D, Enklaar T, Gartner-Rupprecht B, Spangenberg C, Lausch E (2005). Microdeletion and IGF2 loss of imprinting in a cascade causing Beckwith-Wiedemann syndrome with Wilms' tumor.. Nat Genet.

[8] Riccio A, Sparago A, Verde G, De Crescenzo A, Citro V (2009). Inherited and Sporadic Epimutations at the IGF2-H19 locus in Beckwith-Wiedemann syndrome and Wilms' tumor.. Endocr Dev.

[9] Sparago A, Cerrato F, Vernucci M, Ferrero GB, Silengo MC (2004). Microdeletions in the human H19 DMR result in loss of IGF2 imprinting and Beckwith-Wiedemann syndrome.. Nat Genet.

[10] Sparago A, Russo S, Cerrato F, Ferraiuolo S, Castorina P (2007). Mechanisms causing imprinting defects in familial Beckwith-Wiedemann syndrome with Wilms' tumour.. Hum Mol Genet.

[11] Niemitz EL, DeBaun MR, Fallon J, Murakami K, Kugoh H (2004). Microdeletion of LIT1 in familial Beckwith-Wiedemann syndrome.. Am J Hum Genet.

[12] Fitzpatrick GV, Soloway PD, Higgins MJ (2002). Regional loss of imprinting and growth deficiency in mice with a targeted deletion of KvDMR1.. Nat Genet.

[13] Lewis A, Mitsuya K, Umlauf D, Smith P, Dean W (2004). Imprinting on distal chromosome 7 in the placenta involves repressive histone methylation independent of DNA methylation.. Nat Genet.

[14] Mancini-Dinardo D, Steele SJ, Levorse JM, Ingram RS, Tilghman SM (2006). Elongation of the Kcnq1ot1 transcript is required for genomic imprinting of neighboring genes.. Genes Dev.

[15] Mohammad F, Pandey RR, Nagano T, Chakalova L, Mondal T (2008). Kcnq1ot1/Lit1 noncoding RNA mediates transcriptional silencing by targeting to the perinucleolar region.. Mol Cell Biol.

[16] Pandey RR, Mondal T, Mohammad F, Enroth S, Redrup L (2008). Kcnq1ot1 antisense noncoding RNA mediates lineage-specific transcriptional silencing through chromatin-level regulation.. Mol Cell.

[17] Fitzpatrick GV, Pugacheva EM, Shin JY, Abdullaev Z, Yang Y (2007). Allele-specific binding of CTCF to the multipartite imprinting control region KvDMR1.. Mol Cell Biol.

[18] Shin JY, Fitzpatrick GV, Higgins MJ (2008). Two distinct mechanisms of silencing by the KvDMR1 imprinting control region.. EMBO J.

[19] John RM, Ainscough JF, Barton SC, Surani MA (2001). Distant cis-elements regulate imprinted expression of the mouse p57(Kip2) (Cdkn1c) gene: implications for the human disorder, Beckwith–Wiedemann syndrome.. Hum Mol Genet.

[20] Lee MP, DeBaun MR, Mitsuya K, Galonek HL, Brandenburg S (1999). Loss of imprinting of a paternally expressed transcript, with antisense orientation to KVLQT1, occurs frequently in Beckwith-Wiedemann syndrome and is independent of insulin-like growth factor II imprinting.. Proc Natl Acad Sci U S A.

[21] Smilinich NJ, Day CD, Fitzpatrick GV, Caldwell GM, Lossie AC (1999). A maternally methylated CpG island in KvLQT1 is associated with an antisense paternal transcript and loss of imprinting in Beckwith-Wiedemann syndrome.. Proc Natl Acad Sci U S A.

[22] Gabert J, Beillard E, van der Velden VH, Bi W, Grimwade D (2003). Standardization and quality control studies of ‘real-time’ quantitative reverse transcriptase polymerase chain reaction of fusion gene transcripts for residual disease detection in leukemia - a Europe Against Cancer program.. Leukemia.

[23] Algar E, Brickell S, Deeble G, Amor D, Smith P (2000). Analysis of CDKN1C in Beckwith Wiedemann syndrome.. Hum Mutat.

[24] Diaz-Meyer N, Day CD, Khatod K, Maher ER, Cooper W (2003). Silencing of CDKN1C (p57KIP2) is associated with hypomethylation at KvDMR1 in Beckwith-Wiedemann syndrome.. J Med Genet.

[25] Horike S, Mitsuya K, Meguro M, Kotobuki N, Kashiwagi A (2000). Targeted disruption of the human LIT1 locus defines a putative imprinting control element playing an essential role in Beckwith-Wiedemann syndrome.. Hum Mol Genet.

